# Maternity and Child Welfare

**Published:** 1917-01-27

**Authors:** 


### MATERNITY AND CHILD WELFARE.

The appearance of a new monthly journal entitled *Maternity and Child Welfare* synchronises with the announcement that proposals are about to be laid before Parliament under the auspices of the new President of the Local Government Board, Lord Rhondda, designed to promote officially the sort of baby saving which hitherto has been done largely by private effort. In another column of this issue (page 335) will be found such details as are at present available concerning the outlines of Lord Rhondda's Bill. In welcoming both the new monthly journal and the prospect of legislation on this subject, it is a source of legitimate pride to the Editor to recall that as far back as nine years ago he opened the columns of The Hospital to a long series of articles on "Maternity and Mothering."

In the first editorial pronouncement of its existence, *Maternity and Child Welfare* rightly draws attention both to the tremendous success which has attended the Infant Consultation Centres and the Schools for Mothers established in many cities during the last few years, and to some of the factors which have made for this. Much of the popularity of these organisations is "due to the suitability of the means employed to the national temperament, a point too frequently ignored in attempts at quick-change methods of national regeneration. There has been no attempt at compulsion or dragooning; there has been a spirit of helpfulness, of friendliness, of persuasion, and of reason." All of this is very true and very apposite; and is worth bearing in mind when Lord Rhondda's Bill is produced for discussion and criticism. Since the original philanthropic agencies first started Infant Consultations and similar clinics a few years ago, a good many municipalities and other local authorities have taken up the work, either wholly or in co-operation with voluntary agencies. It is avowedly the aim of the President of the Local Government Board to bring the general level of achievement amongst local authorities up to the standard already reached by the most progressive and enlightened. This unexceptionable ambition carries with it a hidden premiss, which may not be altogether easy of achievement—at any rate, by legislative enactment. That is, it implies that every local authority shall be able to lay hands upon medical men as energetic, as able, and as enlightened as those whose pioneer labours have made successes of the clinics already established, many of which are household words to-day for successful results. We confidently anticipate that such will be the case in that halcyon future "after the war"; but equally confidently we assert that it is not likely to be so, at the present moment, with the cream of our younger professional men in the Army and the best of the older ones run off their legs in attending to the wants of the civilian population. Whether Lord Rhondda's plan is to start at once, or to await the return of the Army, remains to be seen: the need is doubtless urgent now, but it would be a pity to prejudice a fine scheme by too great haste in putting it into execution.

The Local Government Board has for some time offered grants in aid of Maternity and Infant Welfare Centres, and has inspected the work of those which have applied for these grants. The Board has, therefore, had ample opportunity of learning how and why these institutions have attained their popularity, and should be enabled in consequence to draw up model schemes for the instruction of those local authorities which have hitherto done nothing. Aptly enough, this very week furnishes a fine example of the value of social work such as this. Dr. Walford, the Medical Officer of Health for Cardiff, reports an infantile death-rate for 1916 in that city of but 88 per 1,000. Although this rate is not by any means as low as it is ideally capable of being, still it is a remarkable achievement when it is realised that never before has Cardiff's rate been below 100 per 1,000, that it was formerly 150 to 160 every year, and that the average for the previous ten years was 117. It is understood that much of the credit of this splendid reduction is due to the Infant Care Department under the management of Dr. Emilie Creaser and her staff of health visitors. What Cardiff has done other towns can do; we trust the example will not be thrown away, and that within a few years it will be the exception, not the rule, to encounter an infantile death-rate of three-figure dimensions in any part of the kingdom.

